# Global research progress of gut microbiota and epigenetics: bibliometrics and visualized analysis

**DOI:** 10.3389/fimmu.2024.1412640

**Published:** 2024-05-13

**Authors:** Siyu Tian, Min Chen

**Affiliations:** ^1^ School of Clinical Medicine, Chengdu University of Traditional Chinese Medicine (TCM), Chengdu, China; ^2^ Hospital of Chengdu University of Traditional Chinese Medicine, Chengdu, China

**Keywords:** gut microbiota, epigenetics, bibliometrics, mechanism, host diseases, gut-brain axis

## Abstract

**Background:**

Gut microbiota is an important factor affecting host health. With the further study of the mechanism of gut microbiota, significant progress has been made in the study of the link between gut microbiota and epigenetics. This study visualizes the body of knowledge and research priorities between the gut microbiota and epigenetics through bibliometrics.

**Methods:**

Publications related to gut microbiota and epigenetics were searched in the Web of Science Core Collection (WoSCC) database. Vosviewer 1.6.17 and CiteSpace 6.1.R2 were used for bibliometric analysis.

**Results:**

WoSCC includes 460 articles from 71 countries. The number of publications on gut microbiota and epigenetics has increased each year since 2011. The USA, PEOPLES R CHINA, and ITALY are at the center of this field of research. The University of California System, Harvard University, and the University of London are the main research institutions. Li, X, Yu, Q, Zhang, S X are the top authors in this research field. We found that current research hotspots and frontiers include short-chain fatty acids (SCFA) play an important role in gut microbiota and epigenetic mechanisms, gut microbiota and epigenetics play an important role in host obesity, diet, and metabolism. Gut microbiota and epigenetics are closely related to colorectal cancer, breast cancer, and inflammatory bowel disease. At the same time, we found that gut microbiota regulates epigenetics through the gut-brain axis and has an impact on psychiatric diseases. Therefore, probiotics can regulate gut microbiota, improve lifestyle, and reduce the occurrence and development of diseases.

**Conclusion:**

This is the first comprehensive and in-depth bibliometric study of trends and developments in the field of gut microbiota and epigenetics research. This study helps to guide the direction of research scholars in their current field of study.

## 1 Introduction

Trillions of species of symbiotic microbes persist in the gastrointestinal tract, collectively known as the gut microbiota, and they are important factors affecting host health and disease (1). The human body and the microbiome are in a state of dynamic balance, and the microorganisms in the gut participate in many physiological functions of the human body, such as fermentation-related food components, vitamin synthesis, and maintenance of intestinal homeostasis (2). In recent years, with the deepening of the study of gut microbiota, it has been found that microbial signals can calibrate the transcriptional program of host cells through epigenetic modification without changing the underlying genetic code. DNA modification, histone modification, and regulation of non-coding RNA are forms of epigenetic changes to which the microbiome is sensitive (3). Studies have found that epigenetics is a key mechanism to regulate the development of host intestinal homeostasis and metabolic disorders. Epigenetic regulation of microbial communities can be influenced by host diet, antibiotic use, infection, etc (4, 5). The effects of microbial metabolites on host health can be achieved by inducing epigenetic modifications, altering DNA methylation, and microRNAs expression (6).

With the in-depth study of the mechanism of gut microbiota, Research on gut microbiota and epigenetics has attracted more and more attention. However, this research area has not been thoroughly dissected using bibliometrics analysis. Bibliometrics analysis allows for quantitative analysis of literature in the field of study, using mathematical and statistical knowledge (7). Bibliometrics analysis can reflect the hot spots, emphases, and frontiers of the research field (8). In order to better grasp the knowledge of this research field, this study focuses on the hot spots, emphases, and trends of gut microbiota and epigenetics research.

## 2 Methods

### 2.1 Literature resources

**Figure 1:**
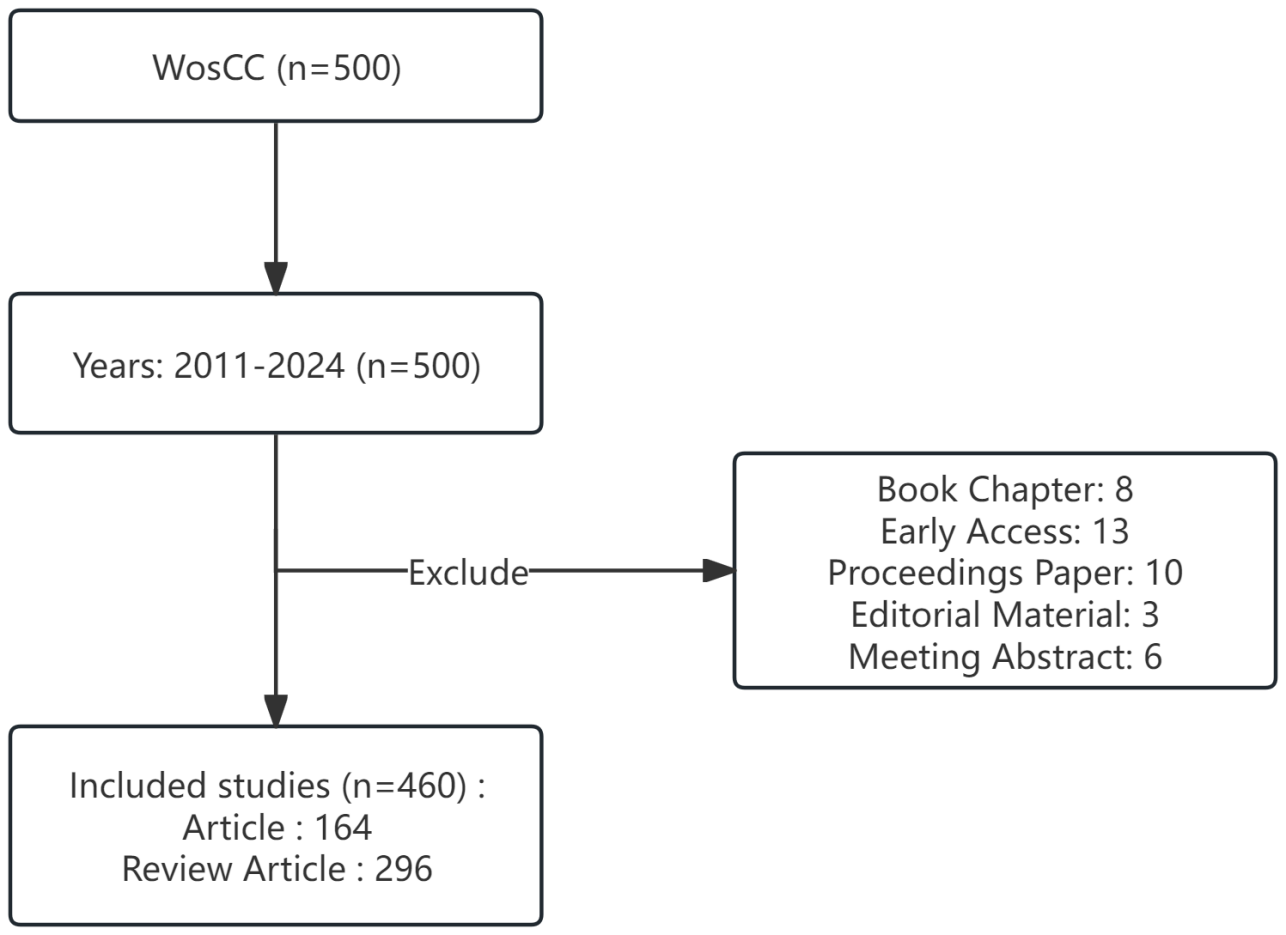
Flow diagram of the included articles.

We searched literature data related to the research field in the Web of Science Core Collection (WoSCC), a multidisciplinary and comprehensive database with a complete citation network (9). The search strategy is presented in **Supplementary Material**, which uses a combination of subject and free words for gut microbiota and epigenetics. The time for a literature search is no limit. The document type is set to Article or Review. The last step is to export and store all the retrieved documents as text files for further bibliometric research. On March 15, 2024, two researchers conducted an independent search of literature data. The complete retrieval process is shown in **Figure 1**.

### 2.2 Literature analysis

We used CiteSpace.6.1.R2, Vosviewer1.6.17, and Microsoft Office Excel 2010 for data analysis and management. Microsoft Office Excel 2010 software can manage data, tally annual publications, and create related tables. In addition, CiteSpace 6.1.R2 creates a visual map that provides a detailed summary analysis of annual publications by number, country, institution, author, keyword, and highly cited article. Vosviewer1.6.17 visualizes highly co-cited literature and co-occurrence of authors. The specific parameter Settings and results of CiteSpace are the same as those of previous Settings (8). Nodes can represent countries and institutions.

## 3 Results

### 3.1 Analysis of annual publications and trends in publications

**Figure 2:**
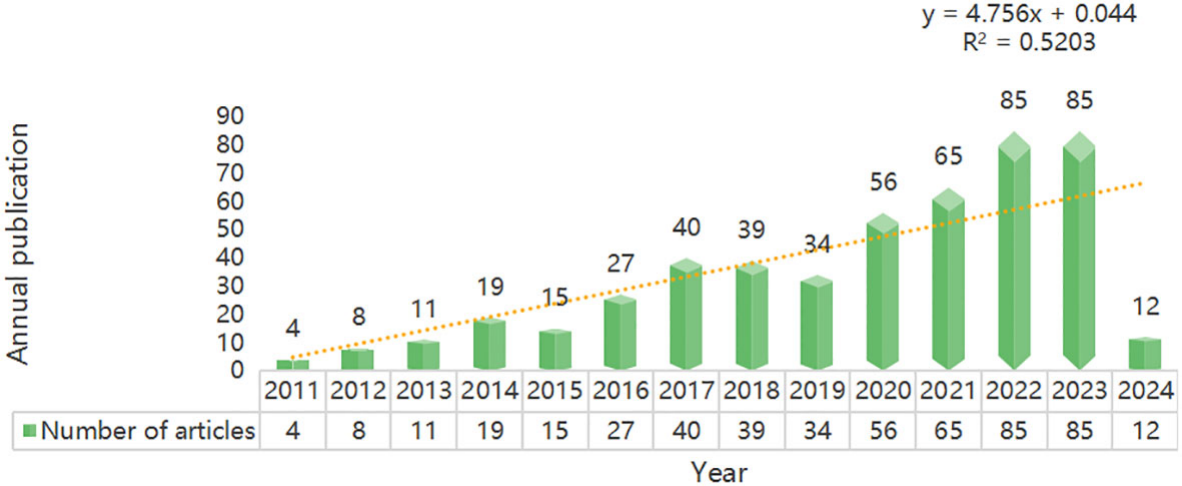
Published trend chart concerning gut microbiota and epigenetics.

Until March 15, 2024, a total of 500 articles have been published in this field, including 164 articles and 296 review articles. Trends in a particular field of research can be measured by annual publications. The analysis shows that the number of papers in this field has increased year by year, from 4 papers in 2011 to a peak in 2022 and 2023 (n=85 papers) (**Figure 2**). This indicates that the field is receiving increasing attention from researchers. In addition, the growth trend model shown in **Figure 2** [coefficient of determination (R^2^) = 0.5203] shows a positive correlation between publication year and publication, which means that the number of annual publications in the field will continue to rise.

### 3.2 Analysis of the trend of countries, institutions, and authors

**Figure 3:**
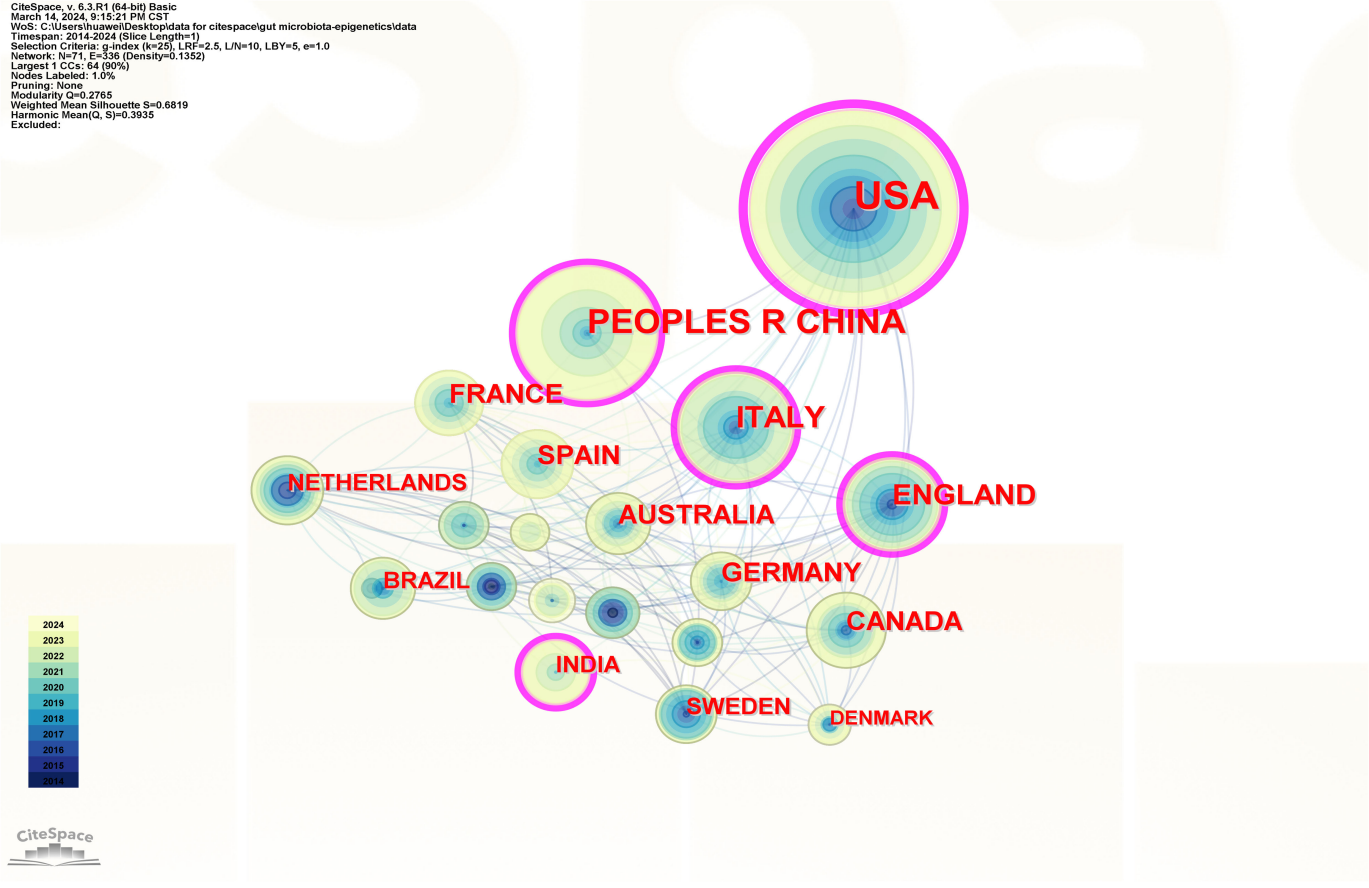
Country/region collaboration network of research on gut microbiota and epigenetics. Created with CiteSpace.

Articles were published in 71 countries/regions. The 71 nodes and 336 links represent countries and cooperation between countries in **Figure 3**. The more a country has published in that area of study, the larger the nodes shown in the graph. If the centrality is greater than 0.1, the purple circle will appear outside the corresponding node on the network map. **Table 1** lists the top 10 countries in terms of the number of published papers and their centrality. The United States published the most papers (168 publications, 32.81%), followed by China (77 publications, 15.04%) and Italy (54 publications, 10.55%), all of which are priority countries for gut microbiota and epigenetics research. Cooperation among countries is positively correlated with centrality. The results show that the United States (0.43), Italy (0.19), the People’s Republic of China (0.18), the United Kingdom (0.16) and India (0.14) are the five countries with the highest centrality.

**Table 1:** Countries/regions, institutions, and authors ranked by publications and centrality.

Item	Rank	Name	Publications	Name	Centrality
Countries/Regions	1	USA	168 (32.81%)	USA	0.43
	2	PEOPLES R CHINA	77 (15.04%)	ITALY	0.19
	3	ITALY	54 (10.55%)	PEOPLES R CHINA	0.18
	4	ENGLAND	40 (7.81%)	ENGLAND	0.16
	5	CANADA	33 (6.45%)	INDIA	0.14
	6	FRANCE	32 (6.25%)	AUSTRALIA	0.10
	7	SPAIN	31 (6.05%)	SPAIN	0.09
	8	AUSTRALIA	30 (5.86%)	SWEDEN	0.09
	9	GERMANY	27 (5.27%)	FRANCE	0.08
	10	NETHERLANDS	20 (3.91%)	NETHERLANDS	0.06
Institutions	1	University of California System	17 (16.83%)	University of California System	0.27
	2	Harvard University	14 (13.86%)	University of London	0.23
	3	University of London	11 (10.89%)	Harvard University	0.18
	4	Baylor College of Medicine	10 (9.90%)	CIBER - Centro de Investigacion Biomedica en Red	0.15
	5	CIBER - Centro de Investigacion Biomedica en Red	9 (8.91%)	Karolinska Institutet	0.11
	6	Harvard Medical School	8 (7.92%)	Brigham & Women’s Hospital	0.10
	7	University System of Ohio	8 (7.92%)	University of Arizona	0.10
	8	Karolinska Institutet	8 (7.92%)	University College Cork	0.09
	9	INRAE	8 (7.92%)	Centre National de la Recherche Scientifique (CNRS)	0.08
	10	Centre National de la Recherche Scientifique (CNRS)	8 (7.92%)	Helmholtz Association	0.08
Authors	1	Li, X	4 (21.05%)	Li, X	0.00
	2	Yu, Q	4 (21.05%)	Yu, Q	0.00
	3	Zhang, S X	4 (21.05%)	Zhang, S X	0.00
	4	He, P F	4 (21.05%)	He, P F	0.00
	5	Dinan, Timothy G	3 (15.79%)	Dinan, Timothy G	0.00

**Figure 4:**
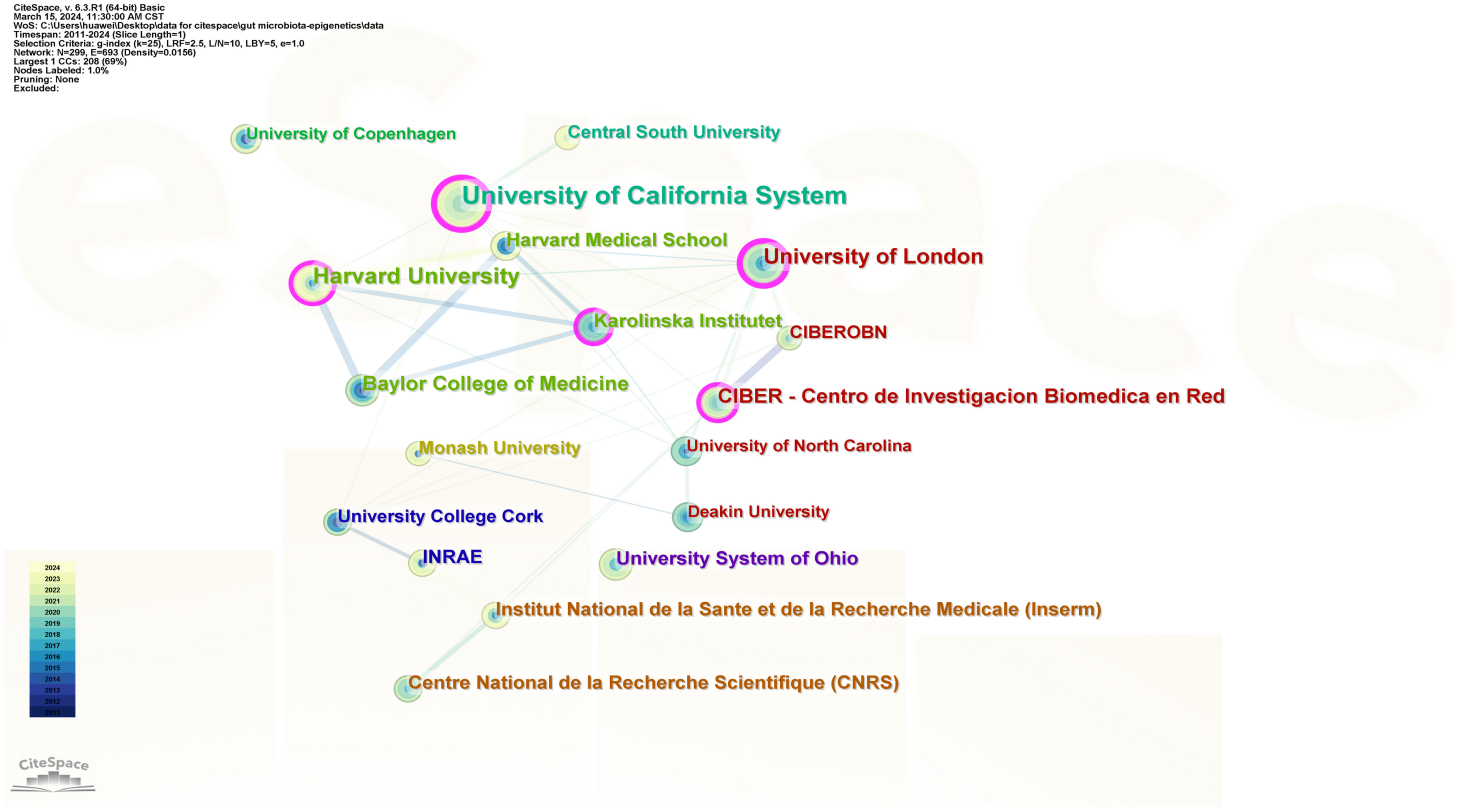
Institution’ collaboration network of research on gut microbiota and epigenetics. Created with CiteSpace.

299 institutions contributed to the field of research. **Figure 4** shows the collaboration between institutions, which includes 299 nodes and 693 connections. From **Table 1**, We found that the top five universities with the highest number of published papers are the University of California System (17 publications, 16.83%), Harvard University (14 publications, 13.86%), the University of London (11 publications, 10.89%), and Baylor College of Science Medicine (10 publications, 9.90%), CIBER-Centro de Investigacion Biomedica en Red (9 publications, 8.91%). The University of California System (0.27), University of London (0.23), Harvard University (0.18), CIBER - Centre for Biomedical Research (0.15), and Karolinska Institutet (0.11) are the top five institutions with the most centricity, representing the most collaboration. The world’s top universities and institutions have made outstanding contributions to the development of the field.

**Figure 5:**
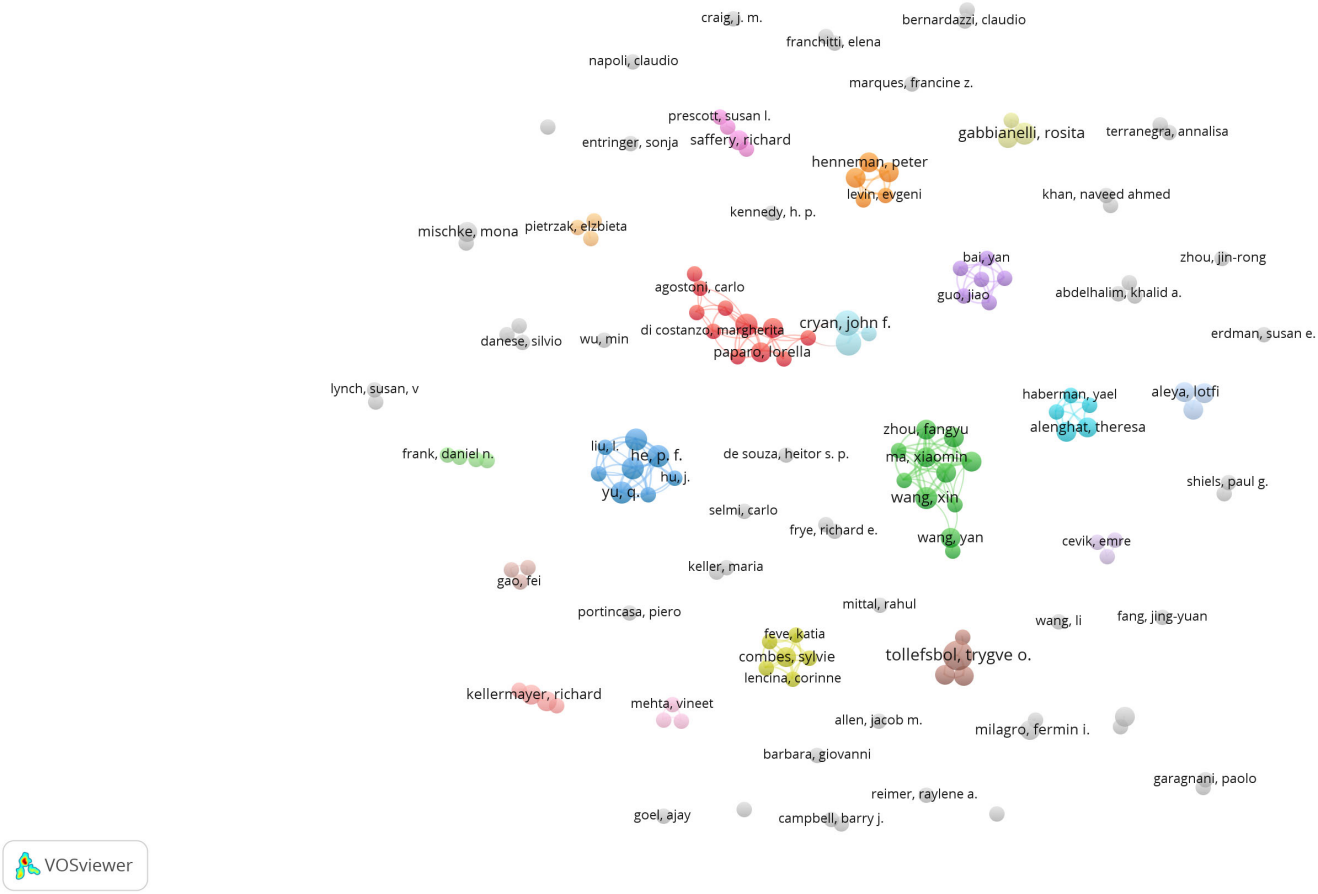
Authors’ collaboration network of research on gut microbiota and epigenetics. Created with VOSviewer.

As shown in **Figure 5**, 293 authors have published papers on gut microbiota and epigenetics. **Table 1** lists the five authors with the highest number of published articles. Four authors, Li, X, Yu, Q, Zhang, S X, He, P F, contributed the most to the number of articles (4 publications per person, 21.05%), followed by Dinan, Timothy G (3 publications, 15.79%). These five authors play important roles in the field of gut microbiota and epigenetics research. The centrality of all authors is 0, indicating that the cooperation between authors still needs to be strengthened.

### 3.3 Analysis of co-cited references

**Figure 6:**
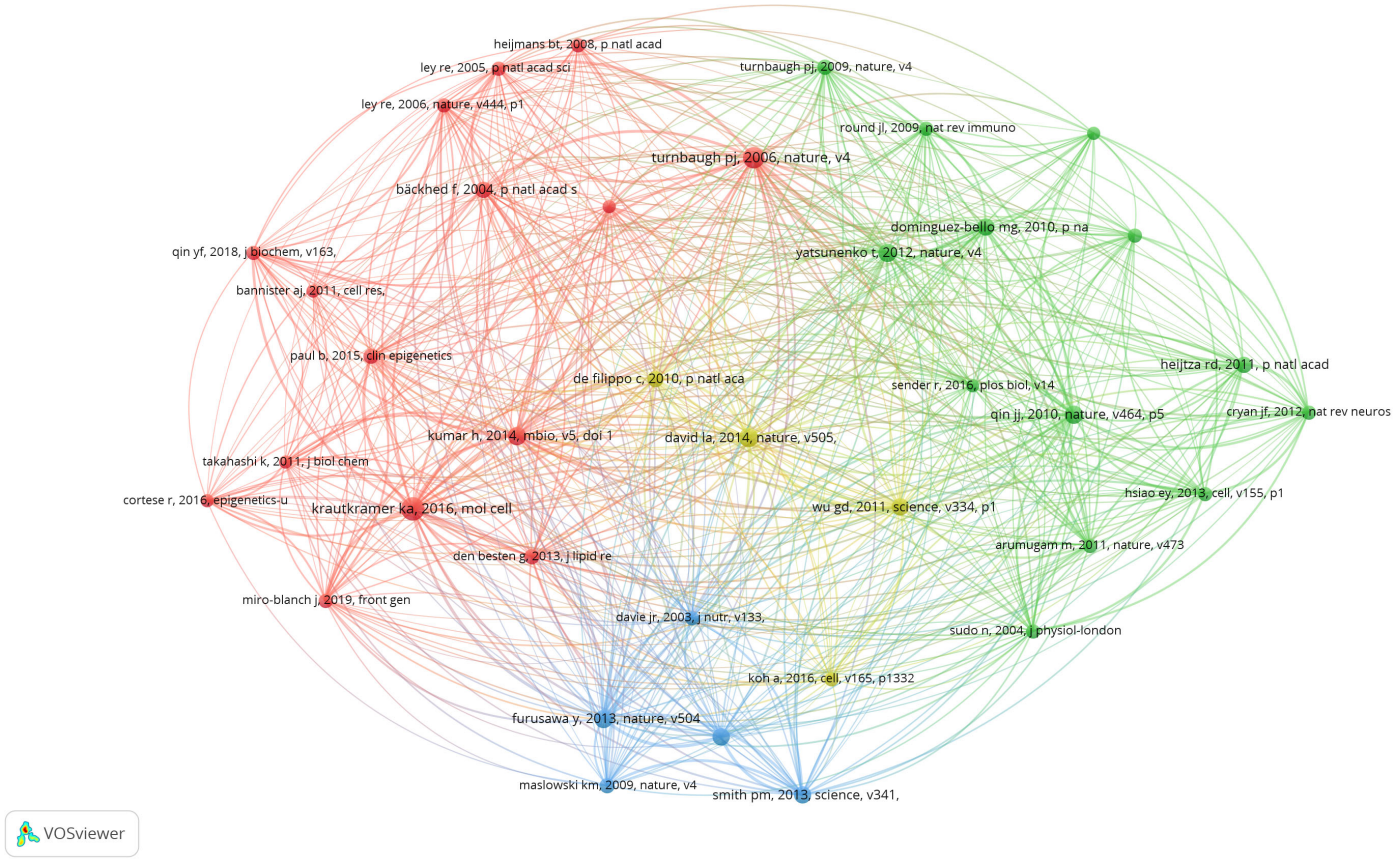
Visualization of a clustering map of co-cited references. Created with VOSviewer.

Co-cited references are those cited collectively by researchers. Through the analysis of co-cited references, VOSviewer visualizes the co-cited references, highlighting common research areas between gut microbiota and epigenetics. According to VOSviewer’s results, a total of 49,507 references were cited in this research area. When the number of citations is reduced to 18, 37 references remain. From **Figure 6**, we can find that the co-cited references are divided into four clusters, corresponding to the four colors in the visualization diagram. The red cluster mainly shows the epigenetic regulation of host metabolism by intestinal microbes, including the epigenetic regulation of host obesity by gut microbiota (10), the interaction between diet and intestinal microbes mediates the epigenetic inheritance of host tissues or diseases (11, 12), and the epigenetic regulation between gut microbiota and host metabolism (13, 14). The literature on green clusters mainly introduces the research on the types and functions of gut microbiota and gene sequencing (15–17). Blue clusters of literature mainly focus on the basic studies on the regulation of intestinal inflammation and immune response by gut microbiota through derivative substances such as butyrate and receptor GPR43 (18–20). The literature in the yellow cluster mainly focuses on the link between diet and gut microbiota, including the key role of short-chain fatty acids (SFCAs) (21–23).

**Table 2** lists the top 10 cited literature, most of which are from the world’s top journals, such as Nature, Science, etc. Therefore, the research on gut microbiota and epigenetics is the current research hotspot and frontier of the scientific community. “Diet-Microbiota Interactions Mediate Global Epigenetic Programming in Multiple Hosts Tissues “is the most widely cited paper in 2016 published in Molecular Cell (12). Among them, Krautkramer et al. proposed that microbial regulation of protein acetylation and methylation in host tissues through diet, as well as short-chain fatty acids fermented by gut microbes, can promote transcriptional responses to host epigenetic programming. In addition, it can be found from the top 10 most-cited papers that most of the cited papers come from high-quality journals such as Nature and Science, which indicates the cutting-edge and innovative nature of this research field. Secondly, most studies in the cited literature focus on how diet and obesity act on the epigenetic inheritance of multiple tissues through gut microbiota, and the regulation of inflammatory immunity by gut microbiota derivatives.

**Table 2:** Top 10 highly co-cited references.

Item	Rank	Title	Citation	Year
Highly co-cited references	1	Diet-Microbiota Interactions Mediate Global Epigenetic Programming in Multiple Host Tissues	24	2016
	2	An obesity-associated gut microbiome with increased capacity for energy harvest	25	2006
	3	Diet rapidly and reproducibly alters the human gut microbiome	26	2014
	4	Gut microbiota as an epigenetic regulator: pilot study based on whole-genome methylation analysis	38	2014
	5	Commensal microbe-derived butyrate induces the differentiation of colonic regulatory T cells	27	2013
	6	A human gut microbial gene catalogue established by metagenomic sequencing	27	2010
	7	Human gut microbiome viewed across age and geography	28	2012
	8	The microbial metabolites, short-chain fatty acids, regulate colonic Treg cell homeostasis	30	2013
	9	Metabolites produced by commensal bacteria promote peripheral regulatory T-cell generation	31	2013
	10	Linking long-term dietary patterns with gut microbial enterotypes	31	2011

The analysis of the top ten cited literature focused on the mechanism between the gut microbiota and epigenetics. In the first ten cited articles, Kimberly A Krautkramer found that short-chain fatty acid (SCFA), a major derivative of the gut microbiota, is able to influence host-related epigenetic phenotypes and is sensitive to host diet (10). Himanshu Kumar’s study found that the gut microbiota, as an epigenetic regulator, in the group dominated by Firmicutes, genes with differential methylation promoters are associated with disease risk, mainly associated with cardiovascular disease, especially with lipid metabolism, obesity, and inflammatory response (29). Patrick M Smith’s study found that short-chain fatty acids, the fermentation products of intestinal microbiota, can regulate Regulatory T cells (Tregs) and thus regulate intestinal inflammation (20). Yukihiro Furusawa’s study found that differentiation of colonic regulatory T cells is induced by butyrate derived from the gut microbiota to improve intestinal inflammation and immune response (19).

### 3.4 Analysis of highly cited literature

**Table 3** shows the top 10 highly cited literature on gut microbiota and epigenetics, most of them come from the world’s top journals and represent the forefront of scientific development. The most cited article is titled “Diet-Microbiota Interactions Mediate Global Epigenetic Programming in Multiple Host Tissues “ (12) indicates that gut mediates the epigenetic state of host tissues and changes in chromatin status to the host and that SCFA influences host epigenetic programming. At the same time, the mechanism research of gut flora and epigenetics also ranked in the top 10.

**Table 3:** Top 10 highly cited references.

Item	Rank	Title	Citation	Year
Highly cited references	1	Diet-Microbiota Interactions Mediate Global Epigenetic Programming in Multiple Host Tissues	30	2016
	2	Diet rapidly and reproducibly alters the human gut microbiome	18	2014
	3	Epigenetic Regulation at the Interplay Between Gut Microbiota and Host Metabolism	18	2019
	4	Crosstalk between the microbiome and epigenome: messages from bugs	16	2018
	5	Gut microbiota as an epigenetic regulator: pilot study based on whole-genome methylation analysis	15	2014
	6	Epigenetic regulation by gut microbiota	15	2022
	7	Metabolites produced by commensal bacteria promote peripheral regulatory T-cell generation	13	2013
	8	Revised Estimates for the Number of Human and Bacteria Cells in the Body	13	2016
	9	Microbiota derived short chain fatty acids promote histone crotonylation in the colon through histone deacetylases	12	2018
	10	The Epigenetic Connection Between the Gut Microbiome in Obesity and Diabetes	12	2020

### 3.5 Analysis of keywords co-occurrence, clustering, burst

**Figure 7:**
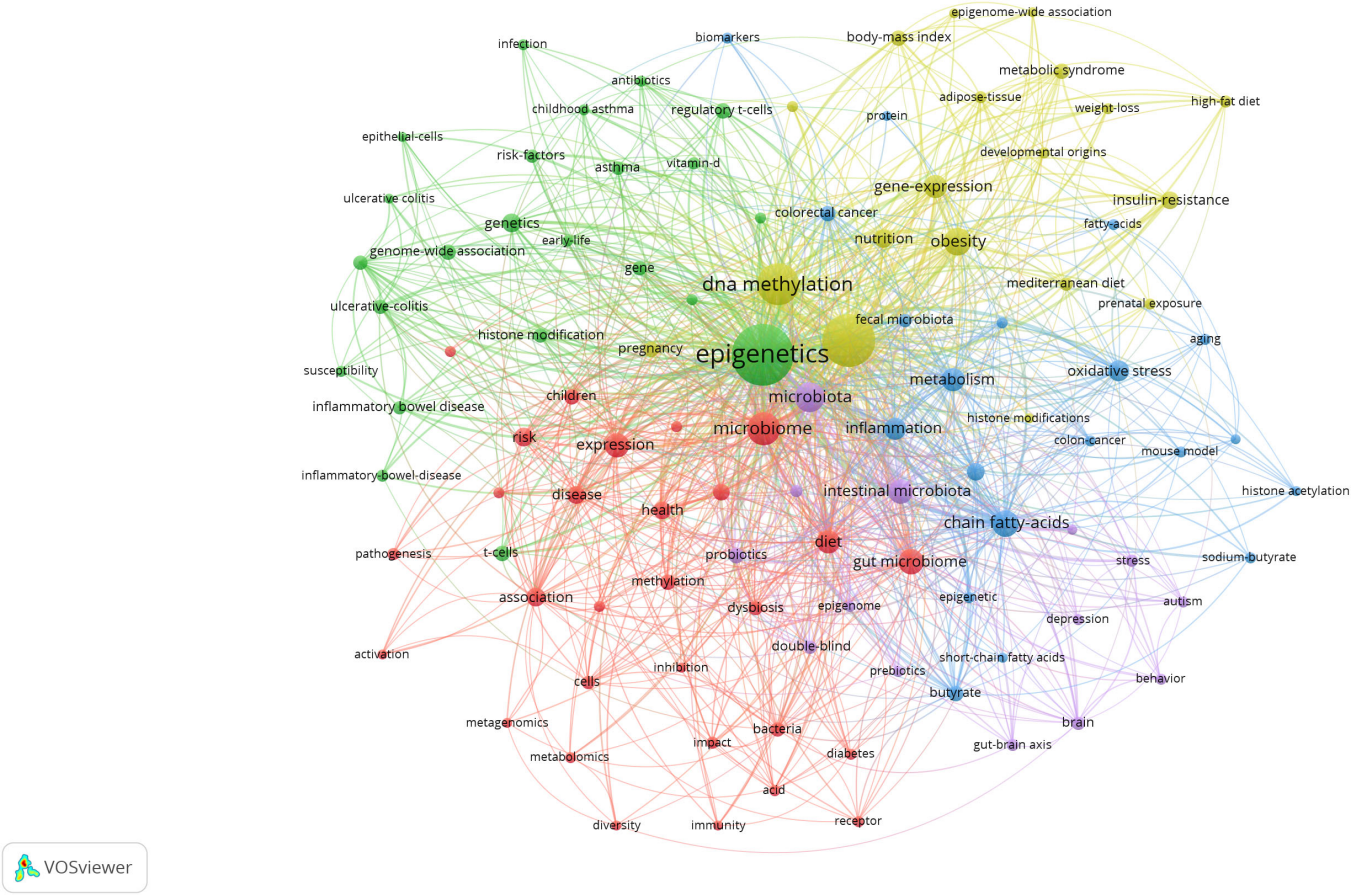
Keyword co-occurrence map of gut microbiota and epigenetics. Created with VOSviewer.

Keyword co-occurrence gives us an idea of the topic and scope of the research field (**Figure 7**). The top 20 keywords in the co-occurrence rate and centrality of gut microbiota and epigenetics from 2011 to 2024 are shown in **Table 4**. “gut microbiota” is the keyword of occurrence frequency, followed by “DNA methylation” and “chain fatty acids”. And more importantly, “gut microbiome”, “gene expression”, “intestinal microbiota”, “expression”, “oxidative stress”, and “colorectal” Keywords such as “cancer” are used more than 30 times, revealing the current research focus and topics in this field. Centrality is positively correlated with the degree of connection between keywords. In **Table 4**, “gut microbiota” is the main intestinal microbiota, followed by “gut microbiome”, “dna methylation”, “association”, and “intestinal microbiota”. These keywords still focus on the link between gut microbiota and epigenetics and the relationship with DNA methylation.

**Table 4:** Top 20 keywords in terms of frequency and centrality.

Rank	Keyword	Frequency	Keyword	Centrality
1	gut microbiota	247	gut microbiota	0.23
2	dna methylation	148	gut microbiome	0.23
3	chain fatty acids	58	dna methylation	0.21
4	gut microbiome	55	association	0.14
5	gene expression	51	intestinal microbiota	0.12
6	intestinal microbiota	51	chain fatty acids	0.11
7	expression	48	gene expression	0.11
8	oxidative stress	33	expression	0.10
9	colorectal cancer	30	colorectal cancer	0.08
10	epigenetics	29	inflammatory bowel disease	0.08
11	association	28	obesity	0.08
12	insulin resistance	25	regulatory t cells	0.07
13	risk	23	health	0.06
14	metabolism	22	inflammation	0.06
15	inflammatory bowel disease	22	mechanisms	0.06
16	health	21	bacteria	0.06
17	inflammation	20	epigenetics	0.05
18	mechanisms	19	insulin resistance	0.05
19	obesity	19	risk	0.05
20	body mass index	19	body mass index	0.04

**Figure 8:**
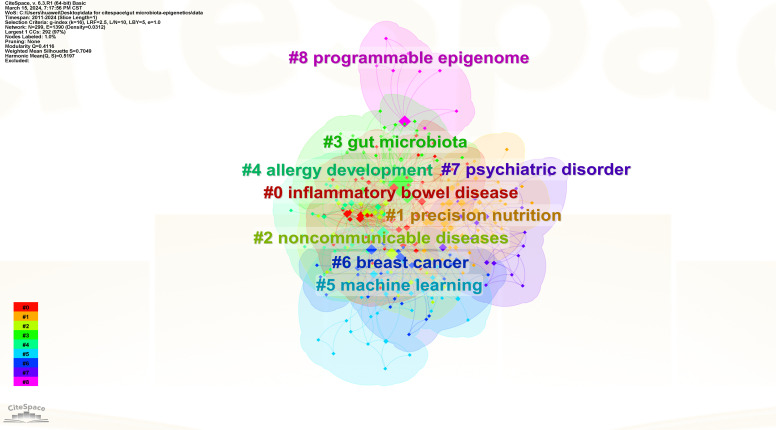
Keyword cluster map of gut microbiota and epigenetics. Created with CiteSpace.

**Figure 9:**
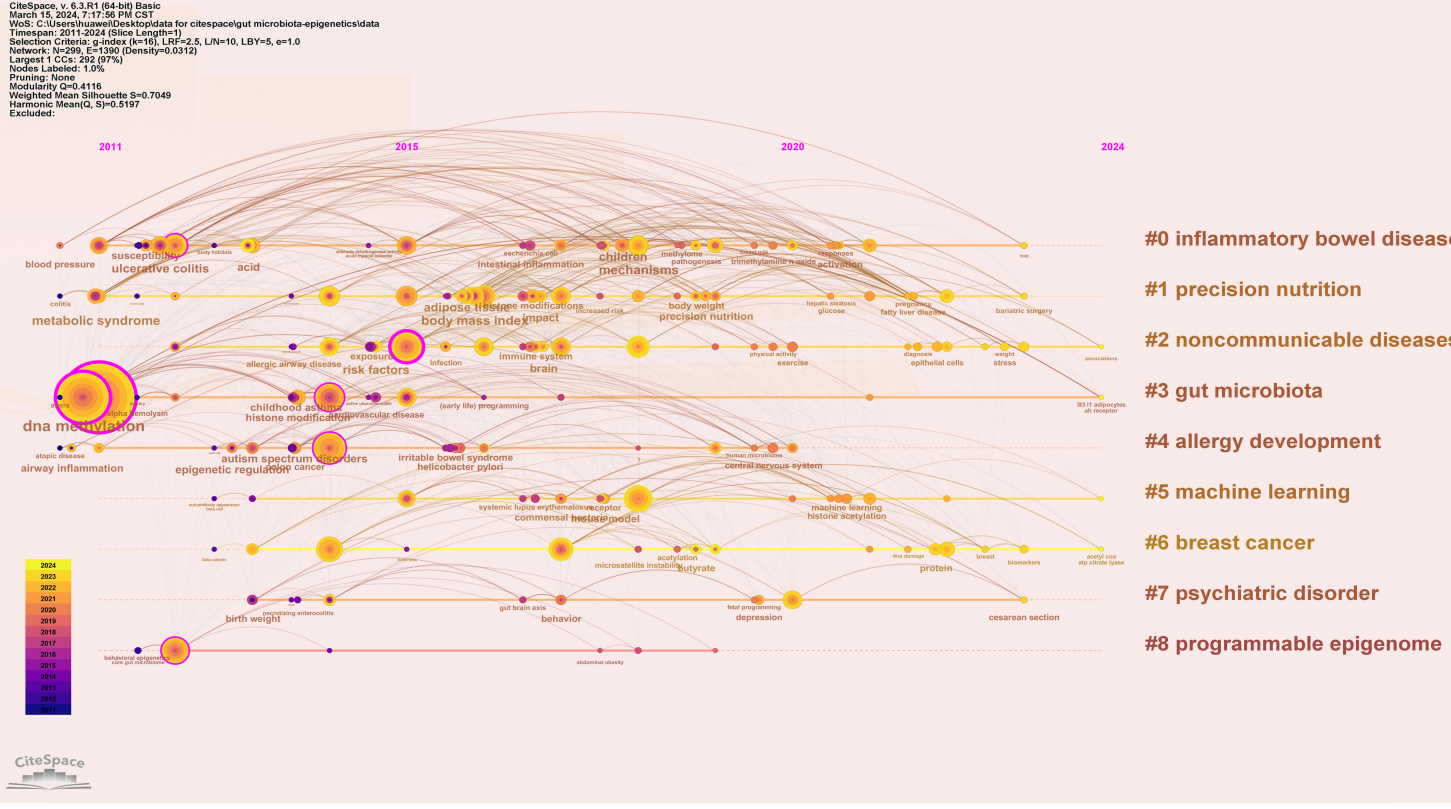
Keyword timeline map of gut microbiota and epigenetics. Created with CiteSpace.

To understand the research frontiers of gut microbiota and epigenetics since 2011, CiteSpace was used to cluster keywords for gut microbiota and epigenetics. Nine clusters are shown in **Table 5**, **Figures 8** and **9**. In general, when Silhouette is greater than 0.5, the clustering effect is reasonable (8). Cluster #0 is labeled “inflammatory bowel disease”, followed by Cluster #1 “Precision nutrition”, Cluster #2 “Noncommunicable diseases”, Cluster #3 “Gut microbiota”, Cluster #4 “Allergy development”, Cluster #5 “Machine learning”, Cluster #6 “Breast cancer”, Cluster #7 “Psychiatric disorder”, Cluster #8 “Programmable epigenome”, representing the forefront of research since 2017.

**Table 5:** Keyword cluster analysis.

Cluster	Size	Silhouette	Mean year	Label (LLR)	Other keywords
0	52	0.572	2017	inflammatory bowel disease	inflammatory bowel disease; gut microbiome; inflammatory bowel diseases; new insight; epigenetic modification; gut microbiota; strain t2; mitochondrial dna; microbial influence; pathogenic role
1	45	0.716	2018	Precision nutrition	gut microbiome; precision nutrition; tackling atherosclerosis; predicting response; current method | gut microbiota; epigenetic regulation; epigenetic mechanism; dna methylation; early life
2	38	0.672	2018	Noncommunicable diseases	developmental origin; noncommunicable diseases; disease concept; cohort profile; neurotypical lymphoblastoid cell line; gut microbiota; gut microbiome; asthma development; recent advance; dna methylation
3	37	0.668	2015	Gut microbiota	gut microbiota; epigenetic regulation; epigenetic factor; gut microbiome; allergic diseases; gut-brain axis; molecular mechanism; functional food; aberrant dna methylation profile; multiple sclerosis patient
4	34	0.741	2015	Allergy development	gut-brain axis; allergy development; emerging role; molecular mechanism; colorectal cancer; gut metabolite; dietary prevention; epigenetic effect; gut maturation; early life
5	31	0.724	2018	Machine learning	brain disorder; multi-omics data analysis; microbiome-mediated epigenetic regulation; machine learning; future direction; wide perspective; glial cell; endocrine cross-talk; review article; gastrointestinal tract
6	28	0.778	2020	Breast cancer	colorectal cancer; breast cancer; short-chain fatty acid; redox homeostasis; host dna methylation change; multi-omics era; intracranial hemorrhage management; central nervous system; practical application; precision oncology
7	18	0.86	2017	Psychiatric disorder	psychiatric disorder; severe mental illness; predictive role; targeting aggression; metabolomic marker; marked methylation change; intestinal gene; preterm neonate; perinatal period; human milk
8	9	0.97	2014	Programmable epigenome	Adult disease; programmable epigenome; peroxisome proliferator-activated receptor; zinc finger protein; connecting homocysteine;intraocular inflammation; new paradigm; inflammatory bowel diseases; adult disease; human health

**Figure 10:**
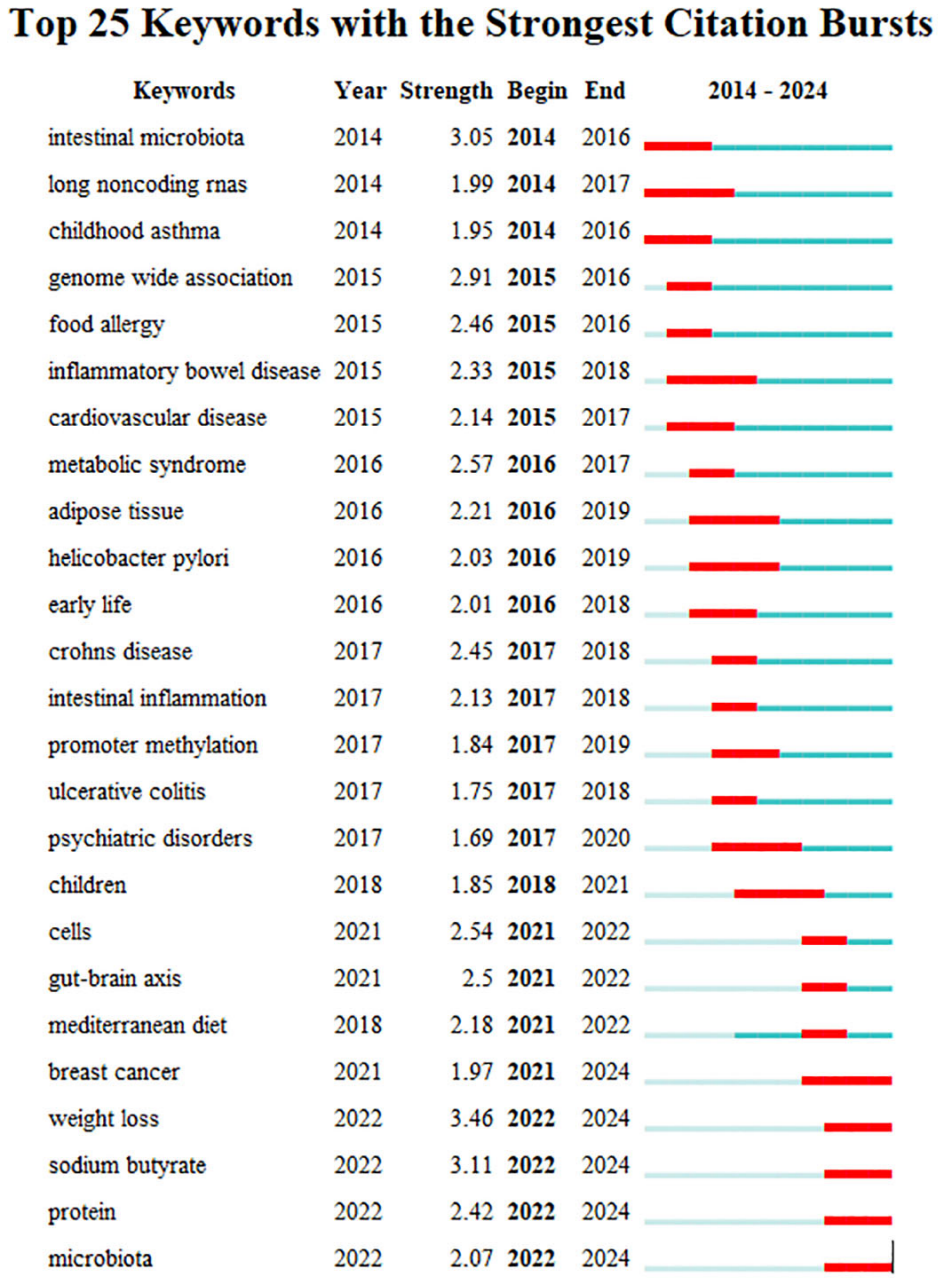
Top 25 keywords with the strongest citation bursts.

Keyword bursts sum up the sudden growth of research content over a period of time, which may indicate future trends in research. **Figure 10** shows the top 25 items with the highest burst intensity in this research subject. The red line in the graph indicates the length of time the keyword bursts. As we observe from the chart, the keyword themes gradually changed from “intestinal microbiota”, “long noncoding RNAs”, “childhood asthma”, “genome-wide association” to the current “breast cancer “, “weight loss”, “sodium butyrate”, “protein” and “microbiota”. This suggests that the correlation between the gut microbiota and epigenetic effects on cancer, metabolism, and mechanisms is the main focus of this research now and in the future.

## 4 Discussion

### 4.1 General information discussion

This study collected all WoSCC data related to the research field to identify research hotspots and frontiers. The number of publications each year has been steadily increasing. With 168 publications, the United States produced the most publications, followed by China and Italy. Because of its very strong economic strength and beneficial policy and scientific support, the United States is the largest country in this field of research. At the same time, although China is a developing country, it has an important position in the field of gut microbiota and epigenetics research.

In specific research areas, collaborations between authors, institutions, and countries can be evaluated using bibliometrics (32). Centrality represents the closeness of cooperation. The top five countries with the highest centrality are the United States, Italy, China, the United Kingdom, and India, meaning that these countries can actively cooperate with different countries. Collaboration between institutions shows that the University of California System, the University of London, Harvard University, CIBER - Centro de Investigacion Biomedica en Red, and Karolinska Institutet cooperate most closely and have the highest central position. Li, X, Yu, Q, and Zhang, S X have published the most papers in this field. However, the centrality of all authors is 0, indicating that there is no cooperation among authors, and cooperation among authors in the field needs to be strengthened. Cooperation among authors requires cooperation in related research fields and policy support from governments. We believe that close cooperation between States, institutions, and authors will help to achieve great progress in this area.

### 4.2 Research focus and hotspot

Bibliometrics analysis can reflect the hot spots and frontiers of this research field. Based on multiple analyses of references and keywords, we found that the hot spots and trends of gut microbiota and epigenetics are related to host metabolism and mechanisms, including obesity, diet, DNA methylation, and the role of SFCAs. In addition, through keyword burst analysis and keyword clustering, it can be seen that scholars have conducted more comprehensive and in-depth research on gut microbiota and epigenetics, and have begun to study the impact of this field on host diseases, such as cancer, inflammatory bowel disease, and mental disorders, as well as research on gut-brain axis theory.

### 4.3 Regulatory mechanisms between gut microbiota and epigenetics

A growing body of evidence supports the interaction of gut microbiota with epigenetic processes. Epigenetic modifications affect host health and disease development by altering the cell’s transcriptional machinery to reprogram the host genome (33). Through bibliometrics analysis, we can learn that the current mechanism between gut microbiota and epigenetics is mainly related to SCFAs, so we will discuss this in detail. The fermentation of complex carbohydrates or starches involves a number of pathways associated with microorganisms (34, 35). After the initial fermentation of carbohydrates in the small intestine, the microbiome ferments it into SFCAs, in which butyrate, propionate, and acetate account for the largest proportion (31). SCFAs can reduce the activity of deacetylase and play an important role in modifying gene expression (36). In one study, SCFAs revealed microbially relevant chromatin modification states and transcriptional reactions, including the regulation of histone acetylation and methylation (12). In addition, propionate and butyrate can promote adipocyte differentiation, which may partially inhibit the effect of histone deacetylase activity (37). SCFAs produced by Akkermansia muciniphila in the mouse ileum can be involved in the expression of histone deacetylase, transcription factors, cellular lipid metabolism, and satiety genes (30). All the above experiments indicate that SCFAs produced by gut microbiota through fermentation have an important influence on host epigenetics.

### 4.4 Effects of interactions between gut microbiota and epigenetic on host metabolism

Based on the results of the bibliometrics analysis, we found that the role of gut microbiota and epigenetics may play an important role in host diet, obesity, and metabolism. The complex interplay between epigenetics, gut microbiota, and diet has important implications for host obesity risk and host metabolic syndrome (6). The study found that the microbial diversity and abundance of obese patients were decreased, the proportion of Bacteroides and Lactobacillus was different, and the methylation levels of FFAR3 gene (FFAR3) and TLR genes TLR4 and TLR2 were decreased. There was a correlation between BMI and methylation of FFAR3 and TLR genes TLR4 and TLR2 (28). In addition, deep sequencing DNA methylation revealed a clear association between gut microbiota and epigenetics (29). One study confirmed that fecal micro-RNA (miRNA) is an important component of the gut microbiome (27). miRNA can mediate bidirectional host-microbial interaction (38). These studies provide insights into the relationship between gut microbes and metabolism-related epigenetics. Based on relevant literature data, further discovery of dietary approaches for beneficial bacterial populations and epigenetic changes in energy homeostasis may have important implications for obesity and metabolism-related clinical manifestations.

### 4.5 Effects of interactions between gut microbiota and epigenetics on host disease

Based on the results of the bibliometrics analysis, we found that the role between gut microbiota and epigenetics may play an important role in inflammatory bowel disease, cancers (colorectal cancer and breast cancer), and psychiatric disorders Research evidence suggests that intestinal microbiota disturbances and alterations in carcinogenic and tumor suppressor genes can cause colorectal cancer (39). The gut microbiota ferments dietary residues, providing energy for the microbiota and ultimately releasing short-chain fatty acids, including butyrate. Butyrate inhibits inflammation and cancer by affecting immunity, gene expression, and epigenetic regulation (40). It was found that microbial fermentation products and activated phytochemicals (such as butyrate and polyphenols) can prevent tumor transformation by inhibiting epigenetic mechanisms such as histone deacetylase (26, 41, 42). The ERα gene and BRCA1 gene, which are strongly associated with breast cancer, have been observed in epigenetic programming (43). The production of butyrate by the gut flora has been shown to activate epigenetic genes in cancer cells such as p21 and BAK (44). However, although gut bacteria can facilitate, epigenetic reprogramming, and contribute to the tumor process, microbiome epigenetic induction of tumor formation has not been proven. Further experiments are needed to confirm this.

Many factors, such as genetic, environmental, intestinal microbiota and immune abnormalities, are related to the occurrence of IBD (45). Genome-wide association studies of IBD identified more than 200 genetic risk loci for IBD, providing important evidence for the role of microorganisms in the pathogenesis of IBD (46). The gut microbiota may regulate epigenetic mechanisms by regulating multiple micronutrients and food components, which may increase the risk of IBD (47).

Multiple evidence suggests that mental illness is related to gut flora and interacts with each other through the gut-brain axis (25, 48, 49). The bidirectional connection between the gut and the brain is called the gut-brain axis. The microbiome is an important part of the triangular conversation (50). Gut microbiota regulates brain function by stimulating neuronal responses or secreting metabolites associated with nerves (51). The gut-brain axis may be involved in the transmission of vagus nerve and hormone signals (52). The gut-brain axis may influence brain functions such as cognition and learning, so targeting a patient’s specific gut flora may reduce symptoms of neurodegenerative diseases (53). The modes of epigenetic regulation include DNA methylation, post-transcriptional histone modification, and gene expression regulation of non-coding RNA (54). DNA methylation is closely related to neurological diseases (24). Gut microbiota can secrete synthetic folic acid, vitamin B12, and choline to produce methyl donors (6-methyltetrahydrofolate) and to form S-adenosine methionine (SAM), which is the main methyl donor in DNA methylation (55). Choline is not only an important nutrient for the brain but also promotes SAM production and is a key methyl donor for DNA and histone methylation (56). The hypothalamic-pituitary-adrenal axis (HPA) is an important communication pathway in the gut-brain axis (51). The normal operation of the HPA axis requires the presence of GR (ligand-activated transcription factor). Studies have found that individuals with genetic abnormalities of the GR gene in the brain are associated with bipolar disorder and schizophrenia (57). Epigenetic modifications do not change the DNA sequence, so the DNA sequence is stable for a long time. The microbiome is capable of modifying the host epigenome via the gut-brain axis and causing visible behavioral or phenotypic changes in the host. Although epigenetic modification changes are more lasting, they are not permanent, so it is possible to restore the gut microbiota and make lifestyle changes (such as sleep, diet, exercise, etc.) through supplementation with probiotics and probiotics, which have important implications for the improvement of conditions such as diabetes, obesity, neurodegenerative diseases, and depression (57).

## 5 Advantages and limitations of research

Visual analysis of bibliometrics can comprehensively display the key points, hot spots, and frontiers of the current research field, and provide researchers with reference research directions. However, there are some limitations to our study. First, we did not search all the databases, which may have led to the omission of literature. In addition, we failed to ensure that every piece of literature fully met the requirements of the study. Finally, the quality of the retrieved articles cannot be completely guaranteed, which will affect the rigor of the analysis.

## 6 Conclusion

This study evaluated and visualized relevant publications on gut microbiota and epigenetics using bibliometrics and visualization analysis. The number of research publications in the field of gut microbiota and epigenetics is increasing every year. The country with the highest number of articles is the United States. The University of California System and Li, X are among the most influential institutions and authors in the field. In addition, our study provides a comprehensive analysis of the research hotspots and research directions of gut microbiota and epigenetics. Based on the bibliometric analysis of gut microbiota and epigenetics, we found that short-chain fatty acids are an important component of the mechanism between gut microbiota and epigenetics. The interaction between gut microbiota and epigenetics play an important role in host obesity, diet, and metabolism. Gut microbiota and epigenetics are closely related to colorectal cancer, breast cancer, and inflammatory bowel disease. At the same time, we found that gut microbiota regulates epigenetics through the gut-brain axis and has an impact on psychiatric diseases. Therefore, probiotics can regulate gut microbiota, improve lifestyle, and reduce the occurrence and development of diseases.

## Data availability statement

The original contributions presented in the study are included in the article/**Supplementary Material**. Further inquiries can be directed to the corresponding author.

## Author contributions

ST: Conceptualization, Data curation, Methodology, Software, Supervision, Visualization, Writing – original draft. MC: Conceptualization, Data curation, Methodology, Software, Writing – review & editing.

## Supplementary material

Supplementary Material Retrieval strategies for gut microbiota and epigenetics.
