# Supplementary material for: Global research progress of gut microbiota and epigenetics: bibliometrics and visualized analysis
Source: Front Immunol. 2024 May 13;15:1412640. doi: 10.3389/fimmu.2024.1412640 (PMC11128553; doi:10.3389/fimmu.2024.1412640)
Supplement: Supplementary Material — Retrieval strategies for gut microbiota and epigenetics. [file DataSheet_1.docx]

# TS = (“Epigenesis, Genetic” OR “Genetic Epigenesis” OR “Epigenetic Processes” OR “Epigenetics Processes” OR “Processes, Epigenetics” OR “Epigenetic Process” OR “Process, Epigenetic” OR “Processes, Epigenetic” OR “Epigenetics”) AND TS = (“Gastrointestinal Microbiome”OR “Gastrointestinal Microbiomes” OR “Microbiome, Gastrointestinal” OR “Gut Microbiome” OR “Gut Microbiomes” OR “Microbiome, Gut” OR“Gut Microflora”OR “Microflora, Gut” OR “Gut Microbiota” OR “Gut Microbiotas” OR “Microbiota, Gut” OR “Gastrointestinal Flora” OR “Flora, Gastrointestinal” OR “Gut Flora” OR “Flora, Gut” OR “Gastrointestinal Microbiota” OR “Gastrointestinal Microbiotas” OR “Microbiota, Gastrointestinal” OR “Gastrointestinal Microbial Community” OR “Gastrointestinal Microbial Communities” OR “Microbial Community, Gastrointestinal” OR “Gastrointestinal Microflora” OR “Microflora, Gastrointestinal” OR “Gastric Microbiome” OR “ Gastric Microbiomes” OR “ Intestinal Microbiome” OR “Microbiome, Gastric” OR “Intestinal Microbiomes” OR “ Microbiome, Intestinal” OR “Intestinal Microbiota” OR “Intestinal Microbiotas” OR “Microbiota, Intestinal” OR “Intestinal Microflora” OR “Microflora, Intestinal” OR “Intestinal Flora” OR “Flora, Intestinal” OR “Enteric Bacteria” OR “Bacteria, Enteric”)
